# A case report of recurrent leiomyosarcoma with chondrosarcoma differentiation in the abdominal wall and a review of the literature

**DOI:** 10.3389/pore.2023.1611109

**Published:** 2023-05-10

**Authors:** Xuan Zuo, Wei L. Wu, Peng Shi, Tian M. Liu, Na Yu, Lei Li

**Affiliations:** ^1^ Department of Pathology, West China Second University Hospital, Sichuan University, Chengdu, China; ^2^ Key Laboratory of Birth Defects and Related Diseases of Women and Children, Ministry of Education, Sichuan University, Chengdu, China

**Keywords:** recurrence, case report, leiomyosarcoma, heterologous differentiation, chondrosarcoma

## Abstract

Leiomyosarcoma with heterologous differentiation is relatively rare. To date, only 19 cases have been reported in the English literature. Heterologous components frequently show histological pleomorphism, while those exhibiting well-differentiated morphology are seldom reported. Here, we report a 34-year-old female diagnosed with leiomyosarcoma and developed abdominal wall recurrence 8 years after primary surgery. The recurrent tumor mainly comprised well-differentiated chondrosarcoma except a single focus of leiomyosarcoma. Due to the rarity and prolonged onset of such a transition, our case provides insight into the understanding of this phenomenon.

## Case report

A 34-year-old woman presented to our hospital with concerns of an abdominal mass with dull pain for several days. Physical examination found a palpable mass measuring 5–6 cm in diameter in the lower abdomen. Computed tomography revealed a multinodular mass measuring 7.6 cm × 4.1 cm × 6.5 cm located in the rectus abdominis muscle with multifocal calcification and uneven enhancement. Serum tumor markers such as CA125 (Cancer Antigen 125), SCC (Squamous Cell Carcinoma Antigen), and CA19-9 (Cancer Antigen 19-9) were all in the normal range. She had a history of “broad ligament leiomyosarcoma” 8 years prior and was treated with radical resection in our hospital. Because recurrence was suspected, she underwent a second surgery for abdominal mass excision. A tough and irregular mass was found beneath the lower abdominal wall during the operation. The mass invaded the subcutaneous fat layer and penetrated through the rectus abdominis muscle. No other intraoperative abnormities were found.

**FIGURE 1:**
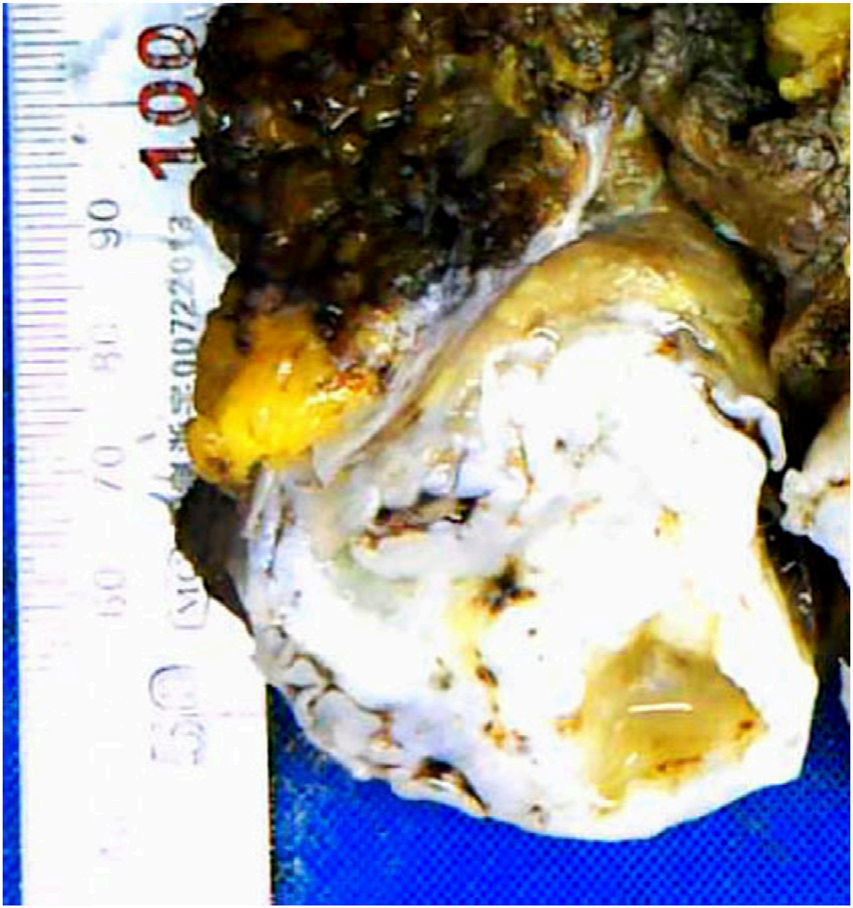
Cartilage-like cut surface of the abdominal wall mass.

Grossly, the tumor had a multinodular appearance in the abdominal muscles below the superficial fascia and intruded into the extraperitoneal adipose tissue. Nodules measuring from 4 to 9 cm were found isolated from each other. All nodules were irregular in shape, with sharp margins and well-demarcated outlines. The cut surfaces were all cartilage-like with a brittle texture, gray to gray‒white color and focal calcification (Figure 1).

**FIGURE 2:**
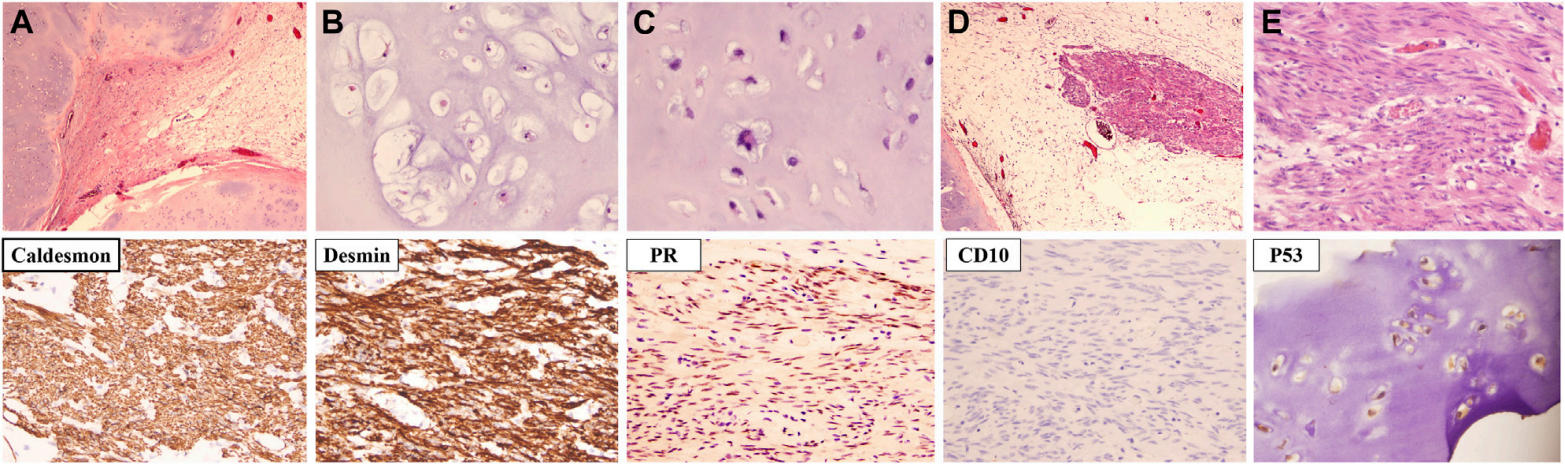
The microscopic characteristics and immunochemical phenotype of the recurrent tumor. **(A)** Cartilage islands were arranged in nodules. **(B)** Chondrocytes were well differentiated with mild to moderate atypia. **(C)** A chondrocyte with bizarre nuclei (indicated by the yellow arrow). **(D)** A bundle of spindle cells was observed next to the cartilage island (indicated by the blue arrow). **(E)** The nucleus of the spindle cell was cigar shaped [Hematoxylin-eosin staining; Magnification: **(A,D)** ×40, **(B–D)** ×200; Immunochemistry staining; Magnification: ×200].

Microscopically, the vast majority of the neoplasm was composed of well-differentiated cartilaginous islands arranged in lobules (Figure 2A). The boundaries were generally smooth. The chondrocytes were uniform in shape with mild to moderate atypia and were unevenly distributed in the cartilage matrix (Figures 2B, C). Aside from the cartilage islands, a single cluster of spindle cells was observed (Figure 2D). The spindle cells were arranged in bundles and featured cigar-shaped nuclei and eosinophilic cytoplasm (Figure 2E). The spindle cells were positive for ER (Estrogen Receptor), PR (Progesterone Receptor), Caldesmon and Desmin, indicating an origin of smooth muscle cells from the genital tract. The strong positivity of P53 in chondrocytes indicated that cartilage islands, although well differentiated, were malignant (Figure 2).

**FIGURE 3:**
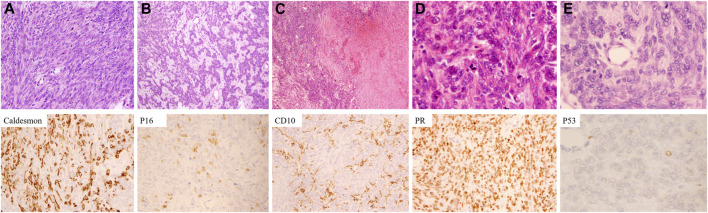
Microscopic characteristics and immunophenotypes of the primary tumor. **(A)** Spindle cells with cigar-shaped nuclei were arranged in a fascicular pattern. **(B)** Epithelioid cells with mild to moderate atypia were lined in a lace-like pattern. **(C)** Coagulative necrosis was frequently seen. **(D)** Mitoses were easily observed. **(E)** Tumor cells exhibited mild to moderate atypia [Hematoxylin-eosin staining: **(A)** ×200. **(**B**)** ×100. **(C)** ×40. **(**D,**E)** ×400. Immunochemistry staining: ×400].

To clarify the relationship between recurrent and primary tumors, we reviewed the histomorphology of the former surgical specimen. The primary tumor was composed of two types of cells: spindle cells and epithelioid cells. Sarcomatoid-like spindle cells were arranged in the fasciculus or vortex with cigar-shaped nuclei and sparse eosinophilic cytoplasm under high magnification (Figure 3A). Epithelioid cells with round to ovoid nuclei and prominent nucleoli arranged in cords or small nests were embedded in the edematous and mucinous matrix (Figure 3B). Marked necrosis could be easily observed under low-power microscopy (Figure 3C). The tumor cells showed overall mild to moderate atypia with active mitosis demonstrating over 10/10 HPF (Figures 3D, E). Such morphological characteristics met the standard for a diagnosis of leiomyosarcoma. It is worth mentioning that no heterologous component was discovered after thorough evaluation.

The final diagnosis was leiomyosarcoma with epithelioid differentiation. The patient then received 4 cycles of APDTIC (EPB, DTIC, DDP) chemotherapy after radical surgery.

The immunoprofiles of primary and recurrent tumor were listed in Table 1. As shown below, tumor cells in both lesions were positive for Vimentin, ER, PR, Caldesmon, SMA (Smooth Muscle Actin), Desmin, and negative for P-CK (pan Cytokeratin). P16 was found patchy in both lesions. The Ki67 index was approximately 25% in the primary neoplasm. Although it was only 2% in the recurrent neoplasm, the increased expression of P53 indicated malignant biological behavior of the recurrent tumor.

**TABLE 1:** The immunophenotypes of primary and recurrent leiomyosarcoma.

Lesion	Vimentin	P-CK	Desmin	SMA	Caldesmon	ER	PR	P53	P16	Ki67 (%)
Primary lesion	+++	–	MF+	MF+	++	+++	++	Single+	F+	35
Recurrent lesion	+++	–	+++	+++	+++	++	F+	+	F+	2

Abbreviations: +, weak positive; ++, moderate positive; +++, strong positive; –, negative; F, focal; MF, multifocal.

Taking into account both the overall medical history and the histopathological characteristics, the abdominal lesion was confirmed to be leiomyosarcoma with chondrosarcoma differentiation. The patient was in good condition after 6 cycles of combined chemotherapy (pharmorubicin, cis-platinum and dacarbazine).

## Discussion

Although leiomyosarcoma is the most common malignant mesenchymal tumor of the uterus, such lesions containing heterologous components are rare, with fewer than 20 cases reported to date (1–9). The clinicopathological characteristics are summarized in Table 2. These tumors mainly affect postmenopausal women. The age of onset is reported to range from 41 to 80 years, with an average age of 53 years. Most patients present with an abdominal mass accompanied by dull pain or vaginal bleeding and irregular menstrual cycles. Most tumors are lobular or nodular, with a median size exceeding 15 cm in diameter (4). The appearance of the cut surface depends on the property and proportion of the heterologous components. Necrosis and hemorrhage are common. These tumors display highly malignant clinical courses and can show remote recurrences (10). Almost all patients experience metastasis or local recurrences (16/19). Lung is the most common recurrence site. The relapse interval varies from 5 months to 9 years. Pathologically, heterologous components can be seen not only in primary lesions but also in metastases. Osteosarcomatous differentiation is most frequent. Most of the heterologous components are poorly differentiated. Notably, such transitions occur mainly in typical leiomyosarcoma, except for one report of a myxoid leiomyosarcoma (1). Whether these changes could occur in pure epithelioid leiomyosarcoma has not yet been reported.

**TABLE 2:** Clinicopathological features of uterine leiomyosarcoma with heterogenous differentiation.

Case	Age	Treatment	Heterologous component	Metastasis/recurrence	Interval before metastasis/recurrence	Prognosis
1	42	S+RA	Chondrosarcoma	Right rectus muscle	3 years	NA
2	45	S	Liposarcoma	Spinal column	6 months	NA
3	53	S	Osteosarcoma	NA	NA	AWD
4	54	S+RA	Liposarcoma, osteosarcoma, chondrosarcoma	Mesentery	8 months	NA
5	50	S	Liposarcoma	Lung, Pelvic cavity	5 months	AWD
6	55	S+CT	Liposarcoma	Pelvic cavity	1 year	NA
7	80	RA+CT	Osteosarcoma, chondrosarcoma, liposarcoma-like area	Lung	11 months	
8	67	SH	Rhabdomyosarcoma	Femur, abductor magnus, lymph node	9 years	AWD
9	41	M+CT	Osteosarcoma	Lung	NA	DOD
10	63	S+CT	Osteosarcoma	Lung, diaphragm, atrium, SVC, kidney	NA	DOD
11	40	S+CT	Osteosarcoma	Colon, retroperitoneum	NA	DOD
12	48	S+CT+RA	Osteosarcoma	Lung, liver, peritoneum, colon, small bowel	NA	DOD
13	55	CT	Osteosarcoma	Pancreas, bone, lung	NA	DOD
14	52	S+CT	Osteosarcoma	Lung, abdomen	NA	AWD
15	61	S+CT+RA	Osteosarcoma	Lung, pleura	NA	DOD
16	53	S	Osteosarcoma	—	—	DOD
17	62	NA	Osteosarcoma	NA	NA	NA
18	43	S+CT	Osteosarcoma	Small bowel	NA	AWD
19	52	S+CT	Osteosarcoma	Liver, small bowel	NA	AWD

Abbreviations: S, surgery; RA, radiation; CT, chemotherapy; SVC, superior vena cava; NA, not available; AWD, alive with disease; DOD, died of disease.

In our case, the patient was initially diagnosed with leiomyosarcoma with epithelioid differentiation and experienced distant recurrence. The recurrent tumor was composed mainly of well-differentiated chondrosarcoma. Misdiagnosis can easily occur regardless of the immunotype and the patient’s medical history. The differential diagnosis mainly depends on the proportion and histologic type of the heterologous component. Cases in which chondrosarcoma and osteosarcoma are the major components, careful sampling and detailed observations are mandatory to discover the smooth muscle component. For leiomyosarcoma with liposarcoma differentiation, the differential diagnosis from dedifferentiated liposarcoma might be challenging. In such cases, molecular tests can provide helpful evidence, as liposarcoma is characterized by the amplification of *MDM2* (11, 12). When two or more unrelated differentiated histology types are confirmed in a single entity, malignant mesenchymoma should be considered (13). However, as most cases can be reclassified, the nomenclature is no longer applied. It is worth noting that sometimes heterologous components may be well differentiated and even mask the nature of the neoplasm. For this reason, a thorough review of the clinical information, careful sampling and skillful pathological examinations are imperative for diagnosis. The overall disease course tends to be aggressive, regardless of the histologic type and the degree of differentiation. More importantly, such tumors respond poorly to combination therapies and could develop late metastases. The mechanism underlying the development of heterologous components is likely to be the reacquisition of stem cell features and the triggering of mesenchymal primordium differentiation (14). An accumulation of similar cases may allow for additional in-depth studies to provide a basis for investigating pathogenetic mechanisms and biological behavior.

The remote metastasis that occurred in our case, although after a long remission interval, urges us to pay sufficient attention to patients who are clinically stable. Regardless the proportion of the heterologous components, the diagnosis of a *de novo* malignancy can only be established if recurrence is completely excluded.

## Data Availability

The original contributions presented in the study are included in the article/supplementary material, further inquiries can be directed to the corresponding author.
